# Effect of IL-6R blockade on plasma lipids and clinical outcomes among hospitalized patients with COVID-19 infection

**DOI:** 10.1016/j.jlr.2024.100568

**Published:** 2024-05-23

**Authors:** Kusha Mohammadi, Mark W. Sleeman, Anita Boyapati, Parnian Bigdelou, Gregory P. Geba, Sergio Fazio

**Affiliations:** Regeneron Pharmaceuticals, Inc., Tarrytown, NY, USA

**Keywords:** COVID-19, CRP, HDL, inflammation, interleukin 6, LDL, lipids, lipoproteins, monoclonal antibody, SARS-CoV-2, triglycerides

## Abstract

Plasma lipid levels are modulated by systemic infection and inflammation; it is unknown whether these changes reflect inflammatory responses or caused directly by pathogen presence. We explored the hypothesis that anti-inflammatory intervention via interleukin 6 receptor (IL-6R) blockade would influence plasma lipid levels during severe infection and evaluated the association of plasma lipid changes with clinical outcomes. Sarilumab (monoclonal antibody blocking IL-6R) efficacy was previously assessed in patients with coronavirus disease 2019 (COVID-19) (NCT04315298). This analysis determined whether strong inflammatory reduction by sarilumab in patients with COVID-19 pneumonia of increasing severity (severe, critical, multisystem organ dysfunction) affected plasma lipid changes between day 1 and day 7 of study therapy. Baseline lipid levels reflected the presence of acute systemic infection, characterized by very low HDL-C, low LDL-C, and moderately elevated triglycerides (TGs). Disease severity was associated with progressively more abnormal lipid levels. At day 7, median lipid levels increased more in the sarilumab versus placebo group (HDL-C +10.3%, LDL-C +54.7%, TG +32% vs. HDL-C +1.7%, LDL-C +15.4%, TG +8.8%, respectively). No significant association between lipid changes and clinical outcomes was observed. In conclusion, severe-to-critical COVID-19 pneumonia causes profound HDL-C depression that is only modestly responsive to strong anti-IL-6R inflammatory intervention. Conversely, LDL-C depression is strongly responsive to IL-6R blockade, with LDL-C levels likely returning to the predisease set point. These results advance our understanding of the complex relationship between serum lipids and infection/inflammation and suggest that HDL-C depression during acute contagious disease is driven by infection and not IL-6–mediated inflammation.

Cytokine-mediated changes in plasma lipid levels accompany acute systemic infection and inflammatory states and flares (1, 2, 3). Levels of total cholesterol (TC), LDL-C, and HDL-C are decreased during acute infection, while triglycerides (TGs), which generally have an inverse relationship with HDL-C levels, typically increase during the acute phase response (1). HDL-C may take part in the response to infection by absorbing and clearing lipophilic antigens (4), such as viral capsid proteins. Furthermore, the drastic decrease in HDL-C during infection may be more than an indicator of disease severity (5), perhaps even contributing to the healing process (6) and predicting clinical outcomes. During recovery, after the acute reaction has passed, plasma lipids and lipoproteins are typically restored to normal levels; however, the depression of plasma HDL-C levels and increase in TG levels can persist in the presence of chronic inflammation, such as HIV and chronic bronchitis (1).

Infection by severe acute respiratory syndrome coronavirus 2 (SARS-CoV-2) causes mild-to-moderate coronavirus disease 2019 (COVID-19) symptoms in most individuals (7), though some develop severe symptoms that require hospitalization and respiratory support (8). Patients with acute COVID-19 infection have also shown that lower HDL-C levels and an inverse relationship between HDL-C and LDL-C levels (up to 2 years before SARS-CoV-2 infection and after COVID-19 diagnosis) have been associated with greater severity and worse clinical outcomes for patients with COVID-19 (9, 10, 11, 12, 13, 14). However, it is not known whether these changes reflect the inflammatory response or are more directly linked to the presence of a pathogenic insult.

Localized inflammation observed during severe COVID-19 has been linked with respiratory distress and is also associated with elevated levels of cytokines, such as interleukin 6 (IL-6) produced by T cells and inflammatory monocytes (15, 16). During the early stages of the pandemic anti-IL-6 therapies, which were already being used as an anti-inflammatory intervention for conditions such as rheumatoid arthritis, were assessed for the treatment of COVID-19 (15).

Sarilumab is a human immunoglobulin G1 monoclonal antibody that selectively binds to the soluble and membrane-bound forms of the IL-6 receptor (IL-6R) and blocks IL-6–mediated signaling (17, 18). An adaptive phase 2/3 study (NCT04315298; Study 2040) investigated the efficacy and safety of sarilumab in hospitalized patients with severe-to-critical COVID-19, but did not establish significant clinical benefits of sarilumab over placebo (19) despite showing a reduction in inflammation. However, post hoc analyses of this study revealed a clinical benefit when sarilumab was coadministered with corticosteroids to reduce inflammation (19).

Here, we conducted a post hoc analysis to assess whether the powerful and rapid reduction in inflammation caused by sarilumab in hospitalized patients with COVID-19 modulated plasma lipid changes over a 7-day period, and whether the effects on lipid changes were associated with clinical outcomes.

## Materials and Methods

### Study design

Details of the clinical study have been reported previously (19). In brief, Study 2040 was an adaptive, phase 2/3, randomized, double-blind, placebo-controlled, multicenter trial of sarilumab in patients hospitalized with severe-to-critical laboratory-confirmed SARS-CoV-2 infection within 2 weeks prior to randomization. Patients were randomized 2:2:1 to receive intravenous sarilumab 200 mg, sarilumab 400 mg, or placebo, and were treated between March 18 and July 2, 2020.

### Serum lipid analyses collection

Per the data collection schedule (19), blood chemistry was performed as a part of the patient’s clinical care up to day 60 (i.e., the end-of-study visit). Limited longitudinal data was available for blood chemistries that included standard lipid panels due to the ongoing pandemic. Research serum samples were collected for a subset of 528 patients. Serum samples collected at predose and at day 7 were evaluated using a standard lipid panel (TC, TGs, HDL-C, and LDL-C) measured by an ADVIA® Chemistry XPT blood chemistry analyzer (Siemens Healthcare). A total of 952 samples collected from 476 patients (n = 54 severe, n = 299 critical, and n = 123 multisystem organ dysfunction [MSOD]) had a full standard lipid panel on day 1 and a day 7 measurement. C-reactive protein (CRP), IL-6, and viral load measurements were previously described (20).

### Clinical outcomes

In this post hoc analysis, we examined changes in calculated LDL-C, HDL-C, TGs, TC, CRP, and IL-6 from day 1 (i.e., the last calculated measurement before the administration of sarilumab) to day 7. If sufficient data were available, subgroups of lipid and inflammatory marker changes by sex, statin use, and corticoid steroid use were also analyzed.

The following outcomes were assessed by subgroups of baseline lipids and changes in lipids to day 7: (1) time to all-cause mortality, defined as the number of days to death of any cause minus the first dose plus one. Patients who were alive were censored up to day 60 or the last follow-up date, whichever came first; (2) time to hospital discharge, defined as the date of discharge minus the first dose date plus 1 day. Patients who were still in hospital were censored at day 29 or the last follow-up date, which ever came earlier; (3) time to clinical status improvement, defined as the number of days to achieve ≥1 point increase in clinical status plus one using a seven-point ordinal scale as per the World Health Organization Master Protocol (V.3.0, March 3, 2020), where 1 = death; 2 = hospitalized, requiring invasive mechanical ventilation or extra corporeal membrane oxygenation; 3 = hospitalized, requiring noninvasive ventilation or high-flow oxygen devices; 4 = hospitalized, requiring supplemental oxygen; 5 = hospitalized, not requiring supplemental oxygen but requiring ongoing medical care (COVID-19–related or otherwise); 6 = hospitalized, requiring supplemental oxygen and no longer requiring ongoing medical care; and 7 = not hospitalized; (4) time to improvement in oxygenation, defined as the number of days from the first dose to the first improvement in oxygenation (peripheral arterial oxygen saturation to the inspired fraction of oxygen ratio [SpO_2_/FiO_2_] > nadir + 50), lasting ≥48 h or until discharge, whichever was first.

### Statistical analyses

All analyses in this post hoc analysis were conducted in the intent-to-treat analysis set, defined as all randomized patients who received at least one dose of study medication. Additionally, patients with or without non-IL-6 therapy (only applicable to phase 3 patients), randomized to intravenous sarilumab 800 mg or who were immunocompromised, were excluded from this post hoc analysis. To explore the standard lipid panel and inflammatory markers in early stages of the COVID-19 infection (i.e., day 1), boxplots were provided to descriptively summarize the distribution. Viral load and quartiles of the lipid panel at day 1 were also descriptively summarized.

The two-tailed Wilcoxon rank sum test was used to test the change from day 1 to day 7 between pooled sarilumab and placebo. The Hodges–Lehmann estimator and 95% confidence interval were provided to quantify the between-group difference in lipids from day 1. *P* values were given to assess nominal strength and were considered nominally significant at the 0.05 alpha level. An unadjusted Cox proportional-hazards model was applied to obtain estimates of the hazard ratio and 95% confidence interval for all clinical outcomes in subgroups of interest: quartiles of baseline lipids and quartiles of change from day 1 to day 7. A test for interaction was used to assess treatment effects within subgroups. Adjusted risk models were considered with a multivariable hazard model including baseline age, sex, race, and changes in lipids at day 7 (TC, TGs, HDL-C, and LDL-C). Covariates were kept in the model if the *P* value for the univariate association with outcomes was <0.1. Analyses were performed in R version 3.6.

### Ethics

The trial was conducted in accordance with the ethical principles of the Declaration of Helsinki and was consistent with the International Conference on Harmonization of Good Clinical Practice Guidelines. Local institutional review boards or ethics committees at each center oversaw the original trial conduct and documentation. All patients provided written informed consent.

## Results

### Baseline demographics and clinical characteristics

Of 1,822 patients in the full phase 2/3 study cohort, consecutive samples taken at days 1 and 7 were available for 26.1% (n = 476) of patients. Of these, 21% were from phase 2 (n = 100) and 79% were from phase 3 (n = 376). All samples were evaluated for a standard lipid panel. The analysis included 363 patients who received sarilumab treatment (200 mg and 400 mg doses pooled) and 113 patients who received placebo. Baseline characteristics were well-balanced across treatment arms (Table 1) and consistent with the full phase 2/3 study cohort.

**Table 1:** Baseline demographic and clinical characteristics

Patient Demographics	*n*	Placebo (n = 113)	Pooled Sarilumab (n = 363)	Overall (N = 476)
Age, years	476			
Mean (SD)		58 (16)	60 (13)	60 (14)
Median (IQR)		60 (48, 71)	61 (53, 69)	61 (51, 70)
Sex, *n* (%)	476			
Male		70 (62)	246 (68)	316 (66)
Female		43 (38)	117 (32)	160 (34)
Race, *n* (%)	476			
White		57 (50)	162 (45)	219 (46)
Black or African American		14 (12)	72 (20)	86 (18)
Asian		1 (0.9)	9 (2.5)	10 (2.1)
American Indian or Alaska Native		2 (1.8)	1 (0.3)	3 (0.6)
Native Hawaiian or other Pacific Islander		0 (0)	1 (0.3)	1 (0.2)
Other		14 (12)	29 (8.0)	43 (9.0)
Not reported		25 (22)	89 (25)	114 (24)
Hypertension, *n* (%)	476			
Yes		60 (53)	170 (47)	230 (48)
Diabetes, *n* (%)	476			
Yes		18 (16)	72 (20)	90 (19)
Phase indication, *n* (%)	476			
Phase 2		20 (18)	80 (22)	100 (21)
Phase 3		93 (82)	283 (78)	376 (79)
COVID-19 status, *n* (%)	476			
Positive		100 (88)	313 (86)	413 (87)
Negative		0	1 (0.3)	1 (0.2)
Missing		13 (12)	49 (13)	62 (13)
COVID-19 severity status, *n* (%)	476			
Severe		13 (12)	41 (11)	54 (11)
Critical		73 (65)	226 (62)	299 (63)
MSOD		27 (24)	96 (26)	123 (26)
Use of steroids, *n* (%)	476			
Yes		31 (27)	93 (26)	124 (26)
Use of statins, n (%)	476			
Yes		11 (10)	32 (9)	43 (9)
CRP, mg/l	460			
Mean (SD)		189 (117)	209 (167)	204 (157)
Median (IQR)		156 (96, 284)	190 (125, 274)	186 (113, 275)
IL-6, pg/ml^a^	435			
Mean (SD)		255 (434)	428 (1,435)	387 (1,273)
Median (IQR)		111 (37, 209)	148 (60, 337)	134 (54, 302)
Cholesterol, mg/dl	476			
Mean (SD)		118 (33)	121 (40)	120 (38)
Median (IQR)		117 (93, 143)	115 (94, 139)	116 (93, 140)
HDL, mg/dl	476			
Mean (SD)		26 (9)	25 (10)	25 (10)
Median (IQR)		25 (19, 32)	25 (19, 31)	25 (19, 31)
LDL, mg/dl	476			
Mean (SD)		71 (27)	72 (35)	71 (34)
Median (IQR)		69 (50, 88)	65 (48, 89)	66 (48, 89)
Triglycerides, mg/dl	476			
Mean (SD)		210 (125)	237 (179)	231 (168)
Median (IQR)		177 (119, 258)	183 (134, 282)	180 (130, 276)

COVID-19, coronavirus disease 2019; CRP, C-reactive protein; IL-6, interleukin 6; IQR, interquartile range; MSOD, multisystem organ dysfunction.

a *P* < 0.05 for treatment versus placebo using Wilcoxon rank-sum test. All other variables were not statistically significant via the Wilcoxon rank-sum test for continuous variables or Chi-square test/fisher exact test (counts <10) for categorical variables.

In this post hoc analysis set, the overall mean (SD) age of patients was 60 (1.4) years (interquartile range, 51–70 years). Most patients were male (66%, n = 316) and predominantly White (46.0%, n = 219). At baseline, 48% were hypertensive (n = 230), 87% were COVID-19 positive (n = 413), and 26% (n = 124) were treated with steroids. Most patients were classified as either critical or with MSOD (n = 299 and n = 123, respectively), and the remaining 11% (n = 54) were classified as having severe COVID-19.

Compared to those given placebo, patients in the pooled sarilumab group had higher median baseline levels of CRP (190 mg/l vs. 156 mg/l) and IL-6 (148 mg/l vs. 111 mg/l). All lipid parameters were similar across treatment arms; the most notable parameter was low mean HDL-C levels at study baseline (25 mg/dl overall).

**Fig. 1:**
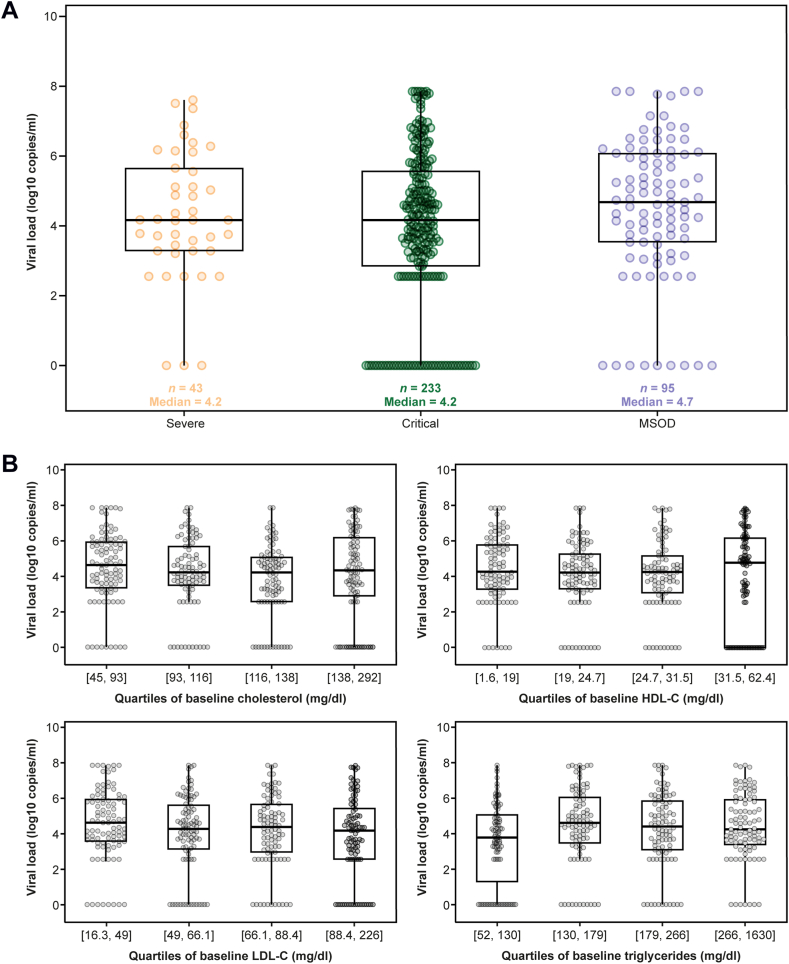
Baseline viral load by (A) COVID-19 severity and (B) quartiles of baseline lipid parameters. MSOD, multisystem organ dysfunction.

Overall, baseline viral load was consistent across severe and critical COVID-19 groups (Fig. 1A). Baseline viral load was also similar across quartiles of baseline lipid parameters (Fig. 1B).

### Changes in inflammatory markers

**Fig. 2:**
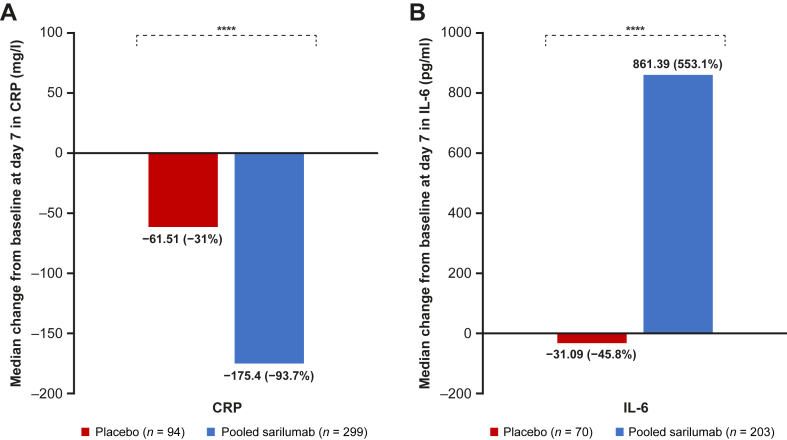
Median change from baseline to day 7 in (A) CRP and (B) IL-6 levels. Of the 476 samples, 393 and 273 patients had day 1 and day 7 measurements for CRP and IL-6, respectively. ∗∗∗∗*P* < 0.0001. CRP, C-reactive protein; IL-6, interleukin-6.

Of the 476 patients included in this post hoc analysis, 83% (n = 392) and 57% (n = 273) had both day 1 and day 7 measurements for CRP and IL-6, respectively. For this particular subgroup analysis, median baseline CRP levels were 156.0 mg/l and 190.0 mg/l for the placebo and sarilumab treatment groups, respectively. Median baseline IL-6 levels were 111.0 pg/ml for the placebo group and 148.0 pg/ml for the sarilumab group. At day 7, CRP levels decreased in patients treated with sarilumab by approximately ∼94% compared with a 31% decrease in the placebo arm (Fig. 2A). In contrast, by day 7, IL-6 increased by approximately 550% in patients treated with sarilumab compared with a ∼46% decrease in the placebo arm (Fig. 2B).

### Changes in lipid parameters

**Fig. 3:**
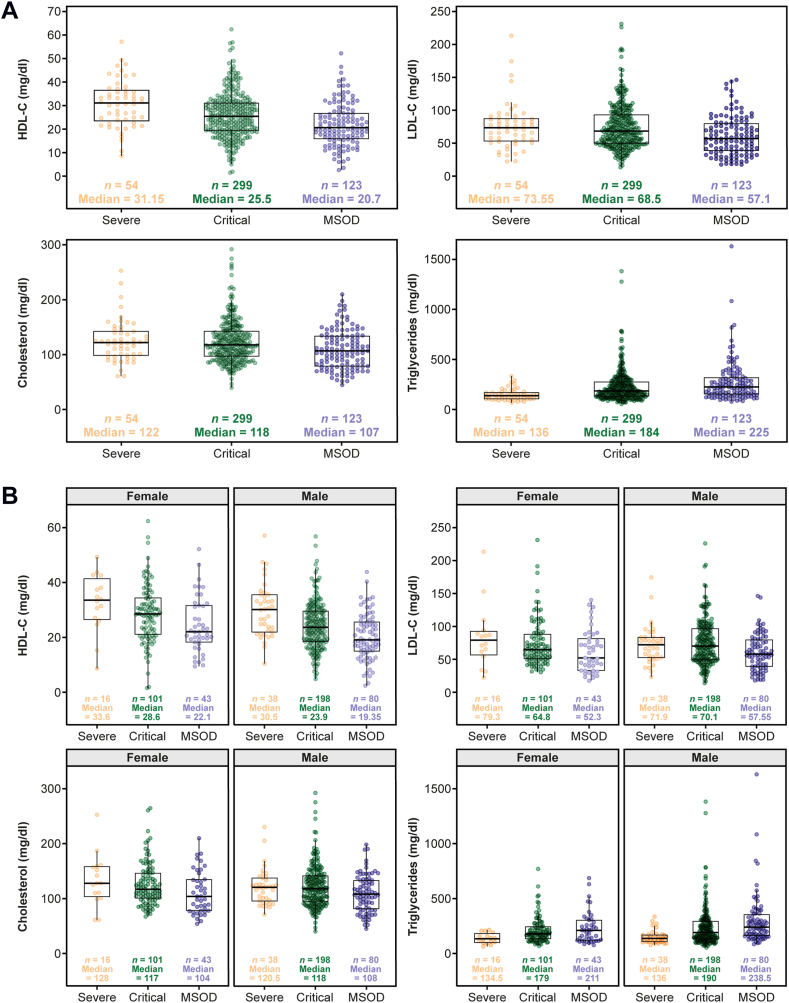
Baseline lipid levels by (A) COVID-19 severity and (B) sex and COVID-19 severity. COVID-19, coronavirus disease 2019; MSOD, multisystem organ dysfunction.

At baseline, HDL-C was low in all COVID-19 severity groups and was progressively lower with more severe COVID-19 presentations (Fig. 3A). Similarly, median LDL-C and TC were low in all COVID-19 severity groups and were lowest in the MSOD group at baseline (Fig. 3A). Median baseline TGs were within the normal range in the groups with less severe forms of COVID-19 but elevated in the critical and MSOD groups (Fig. 3A). A similar trend was observed when baseline lipids were analyzed by sex (Fig. 3B).

**Fig. 4:**
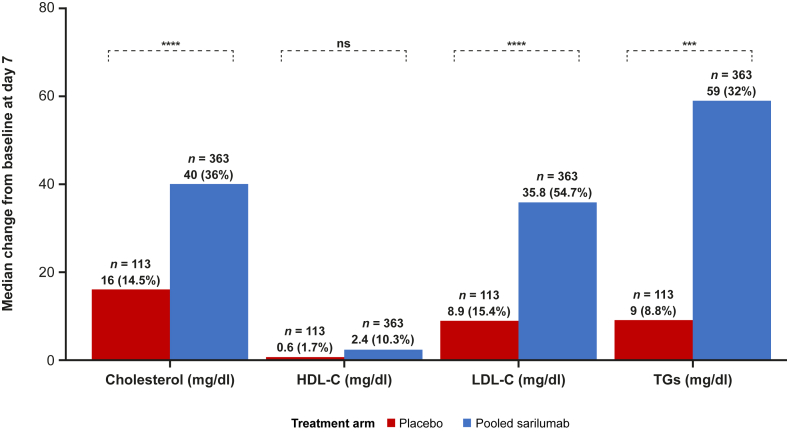
Change in lipid levels from baseline to day 7 by treatment group. ∗∗∗∗*P* < 0.0001 and ∗∗∗*P* < 0.001. ns, not significant; TG, triglyceride.

By day 7, there were significant elevations from baseline in TC, LDL-C, and TGs in patients receiving sarilumab compared with those receiving placebo; a nonsignificant increase in HDL-C was observed (Fig. 4). In subgroup analyses by sex, statin use, and steroid use, median differences between pooled sarilumab and placebo were in the same direction and of a similar magnitude as the main group analysis (Supplemental Fig. S1). Further analyses of the effect of treatment on LDL-C, HDL-C, and TG levels from baseline to day 7 by treatment group are shown in Supplemental Fig. S2A–C, including spaghetti plots of individual changes in lipids from baseline to day 7, individual and mean absolute change, and the proportions of patients with a decrease or increase in lipids.

An analysis of the change in LDL-C with study treatment for those patients with baseline LDL-C levels below a clinically meaningful threshold (<100 mg/dl or <70 mg/dl) and increased above it by day 7 was conducted. From the overall analysis cohort, approximately 5% (6/113) of the patients in the placebo group and approximately 19% (68/363) of the patients in the sarilumab group had LDL-C levels <100 mg/dl at baseline, which increased to >130 mg/dl at day 7 (Supplemental Fig. S2D). Similarly, approximately 1% (1/113) of the placebo group and approximately 10% (35/363) of the sarilumab group had LDL-C levels <70 mg/dl at baseline, which increased to >130 mg/dl at day 7 (Supplemental Fig. S2D).

Median changes from baseline at day 7 for TC and LDL-C were significantly greater in the pooled sarilumab group versus the placebo group for patients with both high and low viral loads at baseline (low: 0–4.29 log copies/ml; high: 4.29–7.85 log copies/ml; Supplemental Fig. S3). In contrast, median increases in TGs were significantly greater for sarilumab versus placebo for patients with low but not high viral loads at baseline (Supplemental Fig. S3). Similarly, median increases in HDL-C with sarilumab versus placebo were only significant for patients with low viral load at baseline (Supplemental Fig. S3).

### Clinical outcomes

**Fig. 5:**
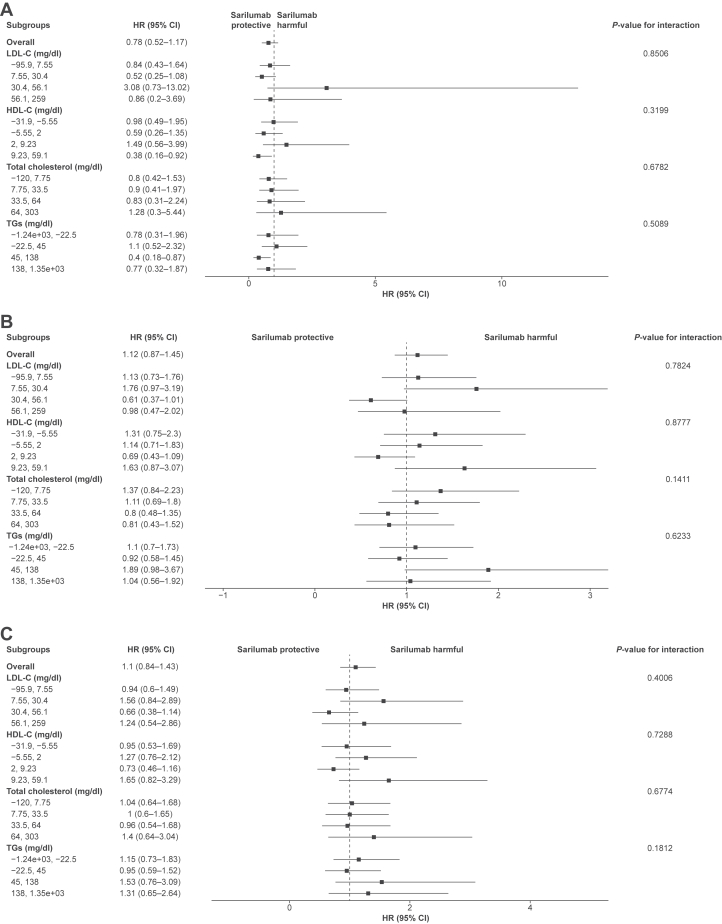
Subgroup analysis for (A) mortality, (B) oxygenation improvement, and (C) clinical improvement by day 60 in change from baseline lipid quartiles. HR, hazard ratio; TG, triglyceride.

Subgroup analysis by day 60 in change from baseline lipid quartiles suggested no treatment benefit with respect to mortality, change in oxygenation status, or clinical improvement (Fig. 5). Additionally, the adjusted hazard models suggested no treatment benefit in any of the four clinical outcomes (Supplemental Table S1).

## Discussion

This post hoc analysis of plasma lipid samples from hospitalized patients with severe-to-critical COVID-19 shows that a marked HDL-C depression is dependent on disease severity and, overall, is not responsive to anti-inflammatory intervention with an IL-6R monoclonal antibody. In contrast, LDL-C depression was much more responsive to anti-inflammatory intervention, and TG levels were increased by the disease and further increased by treatment. Additionally, while sarilumab treatment increased LDL-C and TG levels irrespective of baseline viral load, the increase in HDL-C was more pronounced in those with viral load below the median. Finally, no significant association between lipid changes and clinical outcomes was observed.

Multiple studies have demonstrated decreases in TC, LDL-C, HDL-C, and other lipid parameters in patients with COVID-19 infections (9, 21), as is also common for other infections (1, 22, 23, 24). Greater HDL-C and LDL-C depression is associated with greater severity and worse clinical outcomes for patients with COVID-19 (9, 10, 11, 12, 13, 14). Plasma TG levels tend to be moderately elevated or normal depending on nutritional status and disease severity (9). Furthermore, the ratio of TGs to HDL-C (atherogenic index) predicts mortality in patients with COVID-19 (5, 25). TG levels during COVID-19 are influenced by additional forces affecting the balance between production and clearance of TG-containing lipoproteins (9).

Although COVID-19–associated dyslipidemia is well reported, the underlying mechanisms leading to changes in plasma lipids in patients with COVID-19 remain undefined.

*In vitro* studies have shown that the early stage of SARS-CoV replication, and attachment and internalization within host cells, is dependent upon membrane cholesterol in lipid rafts (26, 27). Disruption of lipid rafts is a possible mechanism by which statins may positively augment the host response to SARS-CoV-2 infection (28). Viruses hijack host lipid metabolism to support their own replication, and reduced plasma levels of cholesterol precursors have been reported in patients with moderate-to-severe COVID-19 (29).

Our findings, that a reduction of inflammation via IL-6R blockade in hospitalized patients with COVID-19 only minimally increased HDL-C levels, suggests that the HDL-C depression observed during acute infection is not linked to the IL-6R–mediated inflammatory state but rather reflects engagement of HDL-C in the immune response, as has been suggested for other viral infections (3). Furthermore, our findings underscore the complexity of the inflammatory process, in which several cytokines and other mediators are involved. For example, interferons, which are induced by viral infections, have been shown to decrease HDL-C levels (30). Additionally, several inflammatory disorders, including rheumatoid arthritis, systemic lupus erythematosus, and psoriasis, are associated with low HDL-C levels and the degree of reduction is related to the severity of the disorder (31). Together, these observations suggest that IL-6 is not the sole mediator of the decrease in HDL-C that occurs during COVID-19 infections. It is also possible that the observations described here may be either specific or more pronounced in the context of COVID-19 compared with other infectious agents. In addition to its role in cholesterol efflux, HDL-C may take part in the response to infection by adsorbing and clearing lipophilic antigens (4, 5, 32). In patients with COVID-19, elevated levels of IL-6 are associated with high levels of lipid peroxidation (33); since HDL-C is eminently prone to oxidative changes (33), sustained elevation of plasma IL-6 resulting from IL-6R blockade may account for the reduced impact of sarilumab on HDL-C levels compared to LDL-C levels.

The lowering of LDL-C levels during initial COVID-19 infection may reflect hepatic metabolic changes resulting from SARS-CoV-2 infection (34). Hepatic LDL receptor transcription has been shown to be stimulated by IL-6, therefore reduced LDL receptor expression resulting from sarilumab treatment is likely to have lowered the uptake of circulating LDL-C by receptor-mediated endocytosis (35). Since IL-6 production does not increase in response to IL-6R inhibition, the large increase in plasma IL-6 levels is likely only due to blockade of the clearance route of the IL6-R (36).

As preexisting dyslipidemia is associated with worse outcomes from SARS-CoV-2 infection, lipid-lowering therapies have been assessed for both the prevention and progression of COVID-19 (28, 37). A retrospective analysis of hospitalized patients with COVID-19 found that the use of statins decreased all-cause mortality (38). It has been postulated that a protective effect of statins may result from their anti-inflammatory effects, possibly via decreasing lipid particle clearance of lipogenic viral particles whose clearance is slowed and half-life prolonged, and/or a direct inhibitory effect on SARS-CoV-2 infectivity (39, 40). However, a meta-analysis of randomized controlled trials observed no benefit in adding statins to COVID-19 treatment (41), a finding further supported by recently published studies (42, 43). In a small study, the use of proprotein convertase subtilisin/kexin type 9 inhibitors was shown to increase survival for 30 patients with COVID-19; however, additional larger studies are required to confirm this finding (44). In addition, patients with long COVID-19 have a higher risk of incident and burden of prevalent dyslipidemia requiring drug therapies (37).

In conclusion, our results show that blockade of IL-6R in hospitalized patients with COVID-19 pneumonia produces only minimal changes in HDL-C levels, suggesting that the viral infection, and not IL-6–mediated inflammation, is the main driver of HDL-C depression. On the contrary, LDL-C levels are affected by both the disease (which lowers them) and the anti-inflammatory therapy (which increases them), whereas TG levels are increased by the disease and further increased by therapy, in an interesting decoupling from HDL-C responses.

## Data availability

Qualified researchers may request access to study documents (including the clinical study report, study protocol with any amendments, blank case report form, and statistical analysis plan) that support the methods and findings reported in this manuscript. Individual anonymized participant data will be considered for sharing once the product and indication has been approved by major health authorities (e.g., Food and Drug Administration, European Medicines Agency, Pharmaceuticals and Medical Devices Agency, etc.), if there is legal authority to share the data and there is not a reasonable likelihood of participant reidentification. Requests should be submitted to https://vivli.org/.

## Supplemental data

This article contains supplemental data.

## Conflict of interest

K. M., M. W. S., A. B., P. B., G. P. G., and S. F. are employees of and stockholders in Regeneron Pharmaceuticals, Inc.

## References

[1] Feingold K.R., Grunfeld C., Feingold K.R., Anawalt B., Blackman M.R., Boyce A., Chrousos G., Corpas E. (2000). Endotext.

[2] Catapano A.L., Pirillo A., Bonacina F., Norata G.D. (2014). HDL in innate and adaptive immunity. Cardiovasc. Res..

[3] Pirillo A., Catapano A.L., Norata G.D., von Eckardstein A., Kardassis D. (2015). High density lipoproteins: From biological understanding to clinical exploitation.

[4] Grao-Cruces E., Lopez-Enriquez S., Martin M.E., Montserrat-de la Paz S. (2022). High-density lipoproteins and immune response: a review. Int. J. Biol. Macromol..

[5] Masana L., Correig E., Ibarretxe D., Anoro E., Arroyo J.A., Jerico C. (2021). Low HDL and high triglycerides predict COVID-19 severity. Sci. Rep..

[6] von Eckardstein A., Nordestgaard B.G., Remaley A.T., Catapano A.L. (2023). High-density lipoprotein revisited: biological functions and clinical relevance. Eur. Heart J..

[7] Yang P., Wang X. (2020). COVID-19: a new challenge for human beings. Cell. Mol. Immunol..

[8] Wu Z., McGoogan J.M. (2020). Characteristics of and important lessons from the coronavirus disease 2019 (COVID-19) outbreak in China: summary of a report of 72 314 cases from the Chinese center for disease control and prevention. JAMA.

[9] Feingold K.R. (2023). The bidirectional interaction of COVID-19 infections and lipoproteins. Best Pract. Res. Clin. Endocrinol. Metab..

[10] Agouridis A.P., Pagkali A., Zintzaras E., Rizos E.C., Ntzani E.E. (2021). High-density lipoprotein cholesterol: a marker of COVID-19 infection severity?. Atheroscler. Plus.

[11] Wei X., Zeng W., Su J., Wan H., Yu X., Cao X. (2020). Hypolipidemia is associated with the severity of COVID-19. J. Clin. Lipidol..

[12] Zinellu A., Paliogiannis P., Fois A.G., Solidoro P., Carru C., Mangoni A.A. (2021). Cholesterol and triglyceride concentrations, COVID-19 severity, and mortality: a systematic review and meta-analysis with meta-regression. Front. Public Health.

[13] Huang S., Zhou C., Yuan Z., Xiao H., Wu X. (2021). The clinical value of high-density lipoprotein in the evaluation of new coronavirus pneumonia. Adv. Clin. Exp. Med..

[14] Hu X., Chen D., Wu L., He G., Ye W. (2020). Declined serum high density lipoprotein cholesterol is associated with the severity of COVID-19 infection. Clin. Chim. Acta.

[15] Narazaki M., Kishimoto T. (2022). Current status and prospects of IL-6-targeting therapy. Expert Rev. Clin. Pharmacol..

[16] Coomes E.A., Haghbayan H. (2020). Interleukin-6 in Covid-19: a systematic review and meta-analysis. Rev. Med. Virol..

[17] Choy E.H., De Benedetti F., Takeuchi T., Hashizume M., John M.R., Kishimoto T. (2020). Translating IL-6 biology into effective treatments. Nat. Rev. Rheumatol..

[18] Khiali S., Rezagholizadeh A., Entezari-Maleki T. (2021). A comprehensive review on sarilumab in COVID-19. Expert Opin. Biol. Ther..

[19] Sivapalasingam S., Lederer D.J., Bhore R., Hajizadeh N., Criner G., Hosain R. (2022). Efficacy and safety of sarilumab in hospitalized patients with coronavirus disease 2019: a randomized clinical trial. Clin. Infect. Dis..

[20] Boyapati A., Wipperman M.F., Ehmann P.J., Hamon S., Lederer D.J., Waldron A. (2021). Baseline severe acute respiratory syndrome viral load is associated with coronavirus disease 2019 severity and clinical outcomes: post hoc analyses of a phase 2/3 trial. J. Infect. Dis..

[21] Feingold K.R., Feingold K.R., Anawalt B., Blackman M.R., Boyce A., Chrousos G., Corpas E. (2000). Endotext.

[22] Alvarez C., Ramos A. (1986). Lipids, lipoproteins, and apoproteins in serum during infection. Clin. Chem..

[23] Khovidhunkit W., Kim M.S., Memon R.A., Shigenaga J.K., Moser A.H., Feingold K.R. (2004). Effects of infection and inflammation on lipid and lipoprotein metabolism: mechanisms and consequences to the host. J. Lipid Res..

[24] Sahin F., Yildiz P. (2013). Distinctive biochemical changes in pulmonary tuberculosis and pneumonia. Arch. Med. Sci..

[25] Turgay Yildirim O., Kaya S. (2021). The atherogenic index of plasma as a predictor of mortality in patients with COVID-19. Heart Lung.

[26] Lu Y., Liu D.X., Tam J.P. (2008). Lipid rafts are involved in SARS-CoV entry into Vero E6 cells. Biochem. Biophys. Res. Commun..

[27] Li G.M., Li Y.G., Yamate M., Li S.M., Ikuta K. (2007). Lipid rafts play an important role in the early stage of severe acute respiratory syndrome-coronavirus life cycle. Microbes Infect..

[28] Talasaz A.H., Sadeghipour P., Aghakouchakzadeh M., Dreyfus I., Kakavand H., Ariannejad H. (2021). Investigating lipid-Modulating agents for prevention or treatment of COVID-19: JACC state-of-the-Art review. J. Am. Coll. Cardiol..

[29] Marcello A., Civra A., Milan Bonotto R., Nascimento Alves L., Rajasekharan S., Giacobone C. (2020). The cholesterol metabolite 27-hydroxycholesterol inhibits SARS-CoV-2 and is markedly decreased in COVID-19 patients. Redox Biol..

[30] Massaro E.R., Borden E.C., Hawkins M.J., Wiebe D.A., Shrago E. (1986). Effects of recombinant interferon-alpha 2 treatment upon lipid concentrations and lipoprotein composition. J. Interferon Res..

[31] Bonacina F., Pirillo A., Catapano A.L., Norata G.D. (2021). HDL in immune-inflammatory responses: implications beyond cardiovascular diseases. Cells.

[32] Trinder M., Boyd J.H., Brunham L.R. (2019). Molecular regulation of plasma lipid levels during systemic inflammation and sepsis. Curr. Opin. Lipidol..

[33] Aparisi Á., Martín-Fernández M., Ybarra-Falcón C., Gil J.F., Carrasco-Moraleja M., Martínez-Paz P. (2022). Dyslipidemia and inflammation as hallmarks of oxidative stress in COVID-19: a follow-up study. Int. J. Mol. Sci..

[34] Kočar E., Režen T., Rozman D. (2021). Cholesterol, lipoproteins, and COVID-19: Basic concepts and clinical applications. Biochim. Biophys. Acta Mol. Cell Biol. Lipids.

[35] Gierens H., Nauck M., Roth M., Schinker R., Schürmann C., Scharnagl H. (2000). Interleukin-6 stimulates LDL receptor gene expression via activation of sterol-responsive and Sp1 binding elements. Arterioscler. Thromb. Vasc. Biol..

[36] Uchiyama Y., Yoshida H., Koike N., Hayakawa N., Sugita A., Nishimura T. (2008). Anti-IL-6 receptor antibody increases blood IL-6 level via the blockade of IL-6 clearance, but not via the induction of IL-6 production. Int. Immunopharmacol..

[37] Xu E., Xie Y., Al-Aly Z. (2023). Risks and burdens of incident dyslipidaemia in long COVID: a cohort study. Lancet Diabetes Endocrinol..

[38] Zhang X.J., Qin J.J., Cheng X., Shen L., Zhao Y.C., Yuan Y. (2020). In-hospital Use of statins is associated with a reduced risk of mortality among individuals with COVID-19. Cell Metab..

[39] Chacko S.R., DeJoy R., Lo K.B., Albano J., Peterson E., Bhargav R. (2021). Association of pre-admission statin use with reduced in-hospital mortality in COVID-19. Am. J. Med. Sci..

[40] Reiner Ž., Hatamipour M., Banach M., Pirro M., Al-Rasadi K., Jamialahmadi T. (2020). Statins and the COVID-19 main protease: in silico evidence on direct interaction. Arch. Med. Sci..

[41] Khalaji A., Behnoush A.H., Alilou S., Rezaee M., Peiman S., Sahebkar A. (2023). Adjunctive therapy with lipid-lowering agents in COVID-19: a systematic review and meta-analysis of randomized controlled trials. Lipids Health Dis..

[42] Hills T.E., Lorenzi E., Berry L.R., Shyamsundar M., Al-Beidh F., Annane D. (2023). Simvastatin in critically ill patients with Covid-19. N. Engl. J. Med..

[43] Eltahan N.H., Elsawy N.H., Abdelaaty K.M., Elhamaky A.S., Hassan A.H., Emara M.M. (2024). Atorvastatin for reduction of 28-day mortality in severe and critical COVID-19 patients: a randomized controlled trial. Respir. Res..

[44] Navarese E.P., Podhajski P., Gurbel P.A., Grzelakowska K., Ruscio E., Tantry U. (2023). PCSK9 inhibition during the inflammatory stage of SARS-CoV-2 infection. J. Am. Coll. Cardiol..

