# Supplementary material for: Effect of IL-6R blockade on plasma lipids and clinical outcomes among hospitalized patients with COVID-19 infection
Source: J Lipid Res. 2024 May 23;65(6):100568. doi: 10.1016/j.jlr.2024.100568 (PMC11237931; doi:10.1016/j.jlr.2024.100568)
Supplement: Supplemental Data [file mmc1.docx]

Supplemental Table 1. Adjusted risk for (A) death, (B) worsening oxygenation status, (C) continued hospitalization, and (D) worsening of clinical status

**A.**

| **Characteristic** | **Univariate** | | | | **Multivariate** | | |
| --- | --- | --- | --- | --- | --- | --- | --- |
|  | ***N*** | **HR** | **95% CI** | ***P*-value** | **HR** | **95% CI** | ***P*-value** |
| Treatment arm | 476 |  |  |  |  |  |  |
| Placebo |  |  |  |  |  |  |  |
| Pooled sarilumab |  | 0.78 | 0.52–1.17 | 0.23 | 0.73 | 0.47–1.13 | 0.2 |
| Age | 476 | 1.05 | 1.03–1.07 | <0.001 | 1.05 | 1.03–1.06 | <0.001 |
| Sex | 476 |  |  |  |  |  |  |
| Female |  | — | — |  |  |  |  |
| Male |  | 0.92 | 0.62–1.35 | 0.66 |  |  |  |
| Race | 476 |  |  |  |  |  |  |
| Other |  | — | — |  |  |  |  |
| White |  | 0.85 | 0.58–1.23 | 0.38 |  |  |  |
| Change from baseline in LDL-C at day 7 | 476 | 1 | 0.99–1.00 | 0.021 | 1.01 | 1.00–1.02 | 0.3 |
| Change from baseline in TG at day 7 | 476 | 1 | 1.00–1.00 | 0.005 | 1 | 1.00–1.00 | <0.001 |
| Change from baseline in HDL-C at day 7 | 476 | 0.98 | 0.96–0.99 | 0.004 | 1.01 | 0.98–1.03 | 0.6 |
| Change from baseline in TC at day 7 | 476 | 1 | 0.99–1.00 | 0.025 | 0.99 | 0.98–1.00 | 0.059 |

**B.**

| **Characteristic** | **Univariate** | | | | **Multivariate** | | |
| --- | --- | --- | --- | --- | --- | --- | --- |
|  | ***N*** | **HR** | **95% CI** | ***P*-value** | **HR** | **95% CI** | ***P*-value** |
| Treatment arm | 476 |  |  |  |  |  |  |
| Placebo |  | — | — |  | — | — |  |
| Pooled sarilumab |  | 1.12 | 0.87–1.45 | 0.38 | 1.09 | 0.83–1.43 | 0.5 |
| Age | 476 | 0.98 | 0.98–0.99 | <0.001 | 0.98 | 0.98–0.99 | <0.001 |
| Sex | 476 |  |  |  |  |  |  |
| Female |  | — | — |  |  |  |  |
| Male |  | 0.99 | 0.79–1.24 | 0.93 |  |  |  |
| Race | 476 |  |  |  |  |  |  |
| Other |  | — | — |  |  |  |  |
| White |  | 1.08 | 0.87–1.34 | 0.49 |  |  |  |
| Change from baseline in LDL-C at day 7 | 476 | 1 | 1.00–1.01 | 0.016 | 1 | 1.0–1.00 | 0.8 |
| Change from baseline in TG at day 7 | 476 | 1 | 1.00–1.00 | 0.051 | 1 | 1.00–1.00 | 0.026 |
| Change from baseline in HDL-C at day 7 | 476 | 1.01 | 1.00–1.02 | 0.008 | 1 | 0.99–1.01 | 0.8 |
| Change from baseline in TC at day 7 | 476 | 1 | 1.00–1.00 | 0.026 | 1 | 1.00–1.01 | 0.2 |

**C.**

| **Characteristic** | **Univariate** | | | | **Multivariate** | | |
| --- | --- | --- | --- | --- | --- | --- | --- |
|  | ***N*** | **HR** | **95% CI** | ***P*-value** | **HR** | **95% CI** | ***P*-value** |
| Treatment arm | 476 |  |  |  |  |  |  |
| Placebo |  | — | — |  | — | — |  |
| Pooled sarilumab |  | 1.02 | 0.79–1.32 | 0.88 | 1.09 | 0.84–1.43 | 0.5 |
| Age | 476 | 0.98 | 0.97–0.99 | <0.001 | 0.98 | 0.98–0.99 | <0.001 |
| Sex | 476 |  |  |  |  |  |  |
| Female |  | — | — |  |  |  |  |
| Male |  | 0.96 | 0.76–1.20 | 0.71 |  |  |  |
| Race | 476 |  |  |  |  |  |  |
| Other |  | — | — |  |  |  |  |
| White |  | 1.13 | 0.91–1.40 | 0.28 |  |  |  |
| Change from baseline in LDL-C at day 7 | 476 | 1 | 1.00–1.00 | 0.053 | 1 | 1.00–1.00 | 0.3 |
| Change from baseline in TG at day 7 | 476 | 1 | 1.00–1.00 | 0.006 | 1 | 1.00–1.00 | 0.071 |
| Change from baseline in HDL-C at day 7 | 476 | 1.01 | 1.00–1.02 | 0.005 | 1 | 0.99–1.02 | 0.5 |
| Change from baseline in TC at day 7 | 476 | 1 | 1.00–1.00 | 0.17 |  |  |  |

**D.**

| **Characteristic** | **Univariate** | | | | **Multivariate** | | |
| --- | --- | --- | --- | --- | --- | --- | --- |
|  | ***N*** | **HR** | **95% CI** | ***P*-value** | **HR** | **95% CI** | ***P*-value** |
| Treatment arm | 476 |  |  |  |  |  |  |
| Placebo |  | — | — |  | — | — |  |
| Pooled sarilumab |  | 1.1 | 0.84–1.43 | 0.5 | 1.09 | 0.84–1.43 | 0.5 |
| Age | 476 | 0.98 | 0.97–0.99 | <0.001 | 0.98 | 0.98–0.99 | <0.001 |
| Sex | 476 |  |  |  |  |  |  |
| Female |  | — | — |  |  |  |  |
| Male |  | 0.93 | 0.73–1.17 | 0.52 |  |  |  |
| Race | 476 |  |  |  |  |  |  |
| Other |  | — | — |  |  |  |  |
| White |  | 1.05 | 0.84–1.31 | 0.69 |  |  |  |
| Change from baseline in LDL-C at day 7 | 476 | 1 | 1.00–1.01 | 0.005 | 1 | 1.00–1.00 | 0.3 |
| Change from baseline in TG at day 7 | 476 | 1 | 1.00–1.00 | 0.001 | 1 | 1.00–1.00 | 0.071 |
| Change from baseline in HDL-C at day 7 | 476 | 1.02 | 1.01–1.02 | <0.001 | 1 | 0.99–1.02 | 0.5 |
| Change from baseline in TC at day 7 | 476 | 1 | 1.00–1.00 | 0.17 |  |  |  |

*P*-values <0.1 are kept in the model.

HR, hazard ratio; TC, total cholesterol; TG, triglyceride.

Supplemental Figure 1. Subgroup analyses of change in (A) LDL-C, (B) HDL-C, and (C) TG by sex, statin use, and corticosteroid use

**A.**
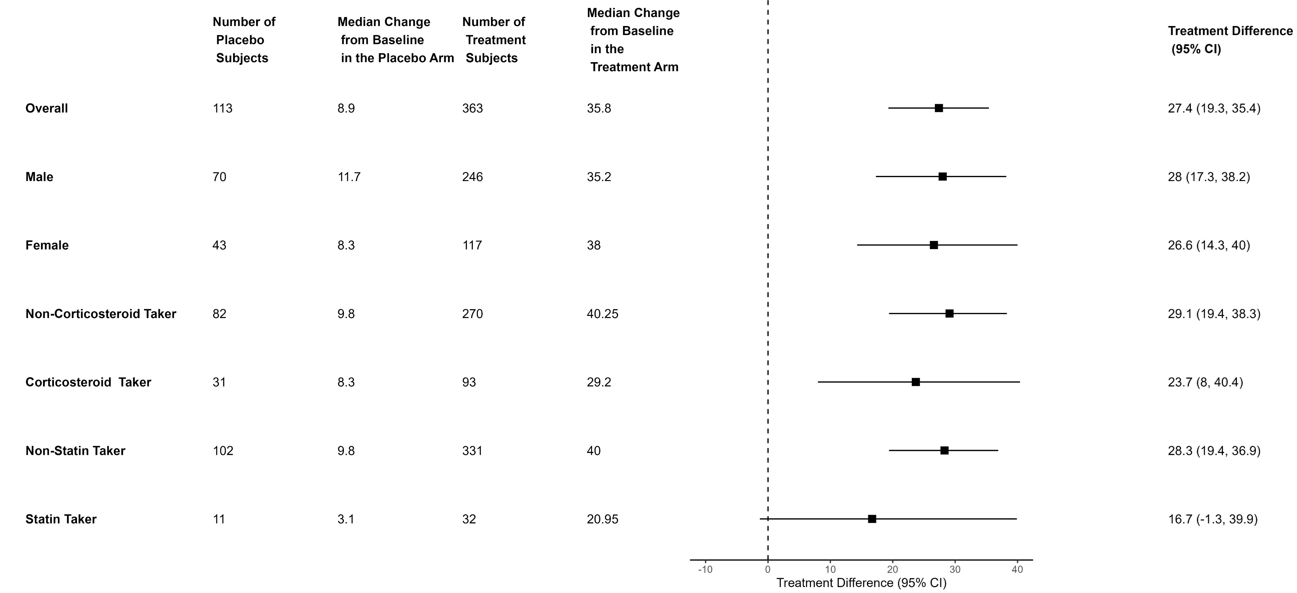


**B.**
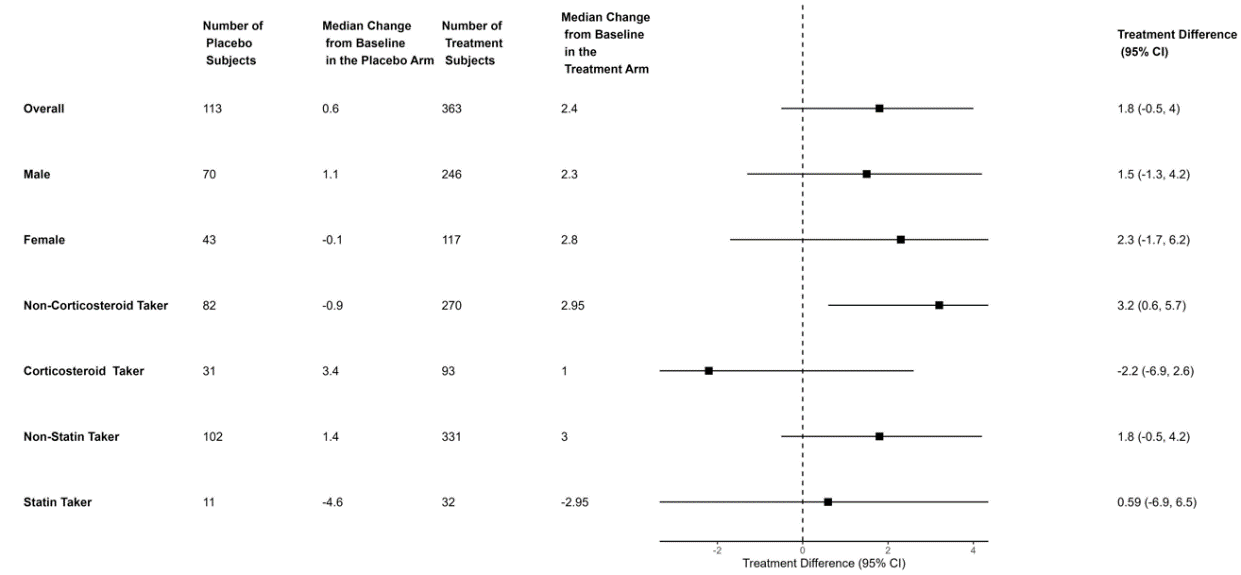


**C.**
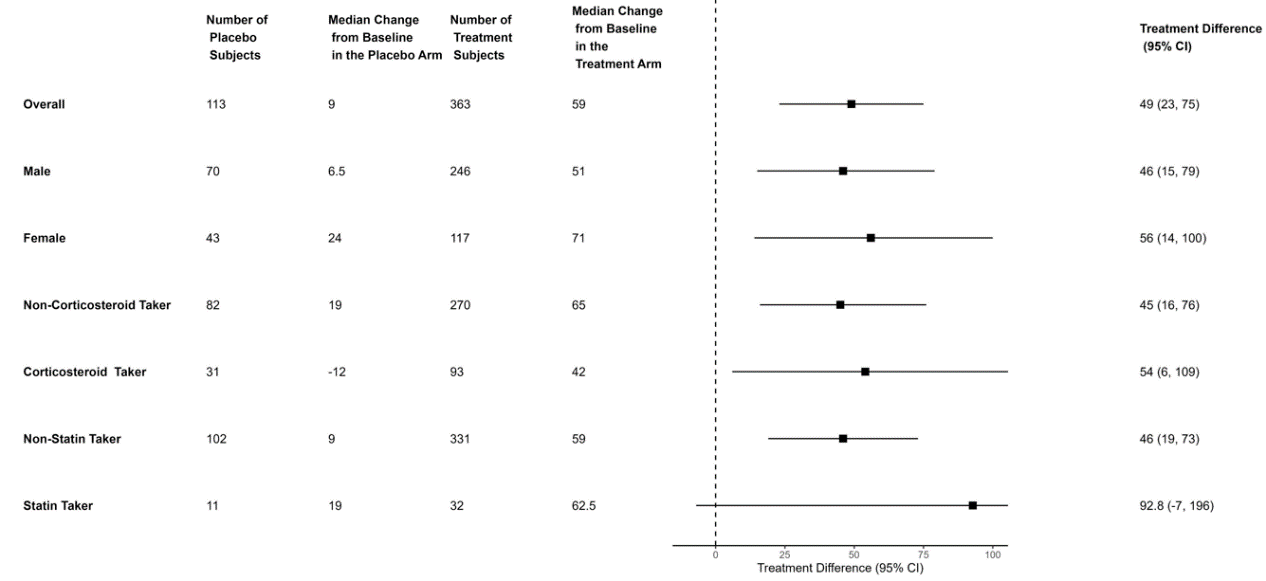


TG, triglyceride.

Supplemental Figure 2. Effect of study treatment with sarilumab or placebo on (A) LDL-C, (B) HDL-C, and (C) TG in all patients, plus (D) LDL-C in patients whose LDL-C was below the clinically meaningful threshold at day 1 and above the threshold at day 7^a^

**A.**
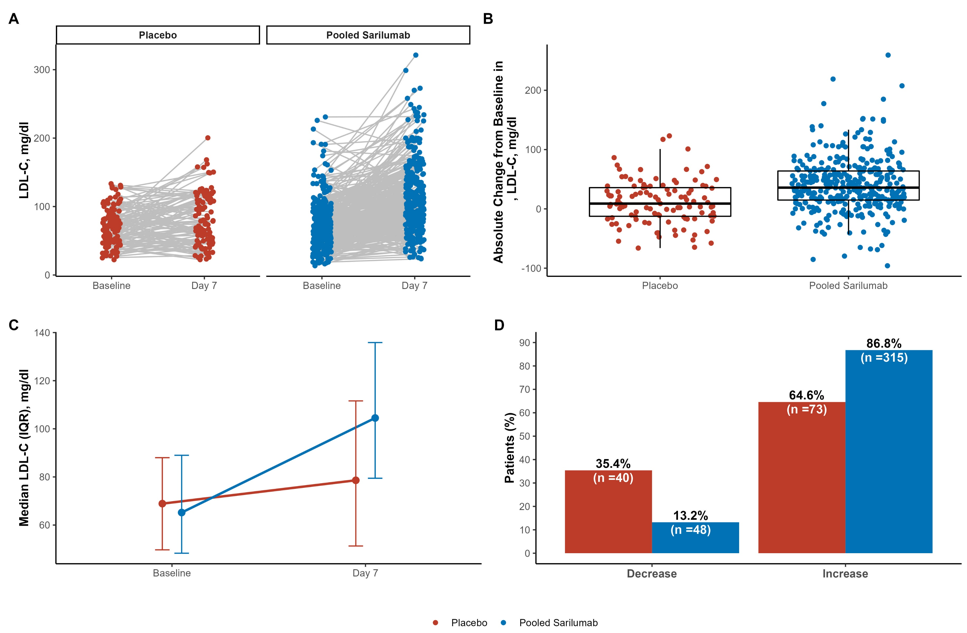


**B.**
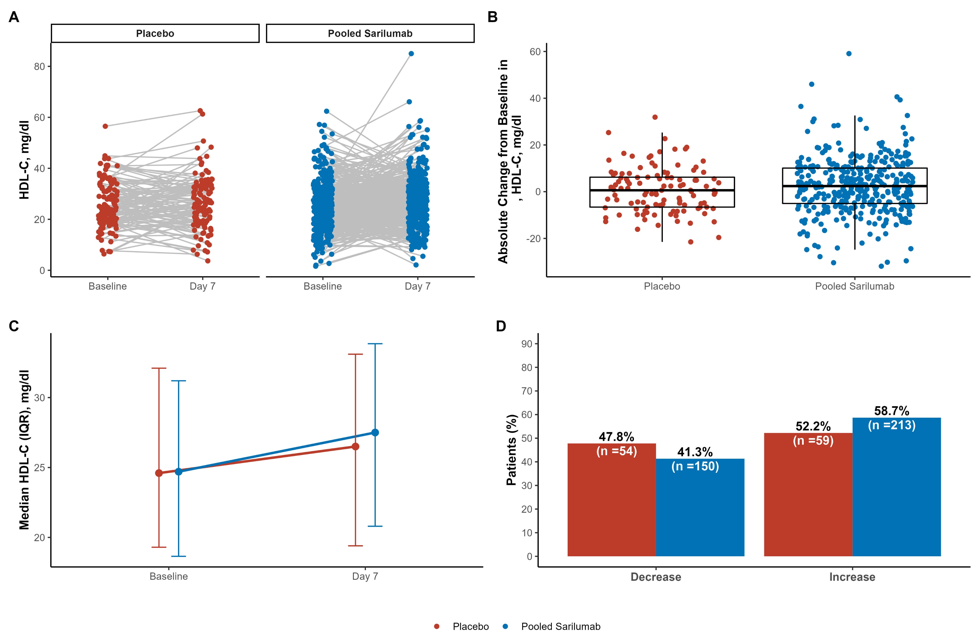


**C.**
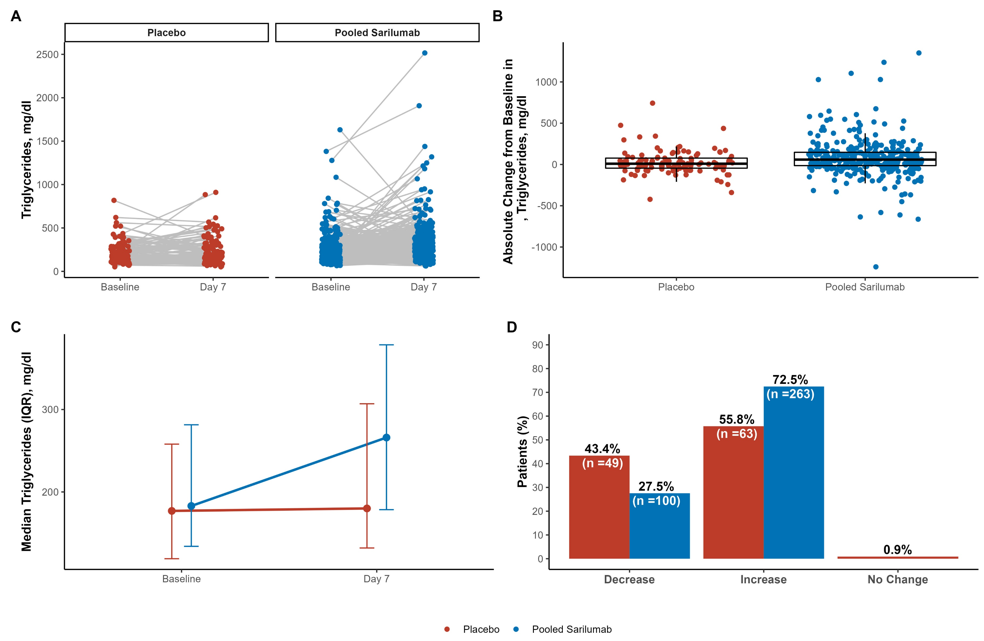


**D.**
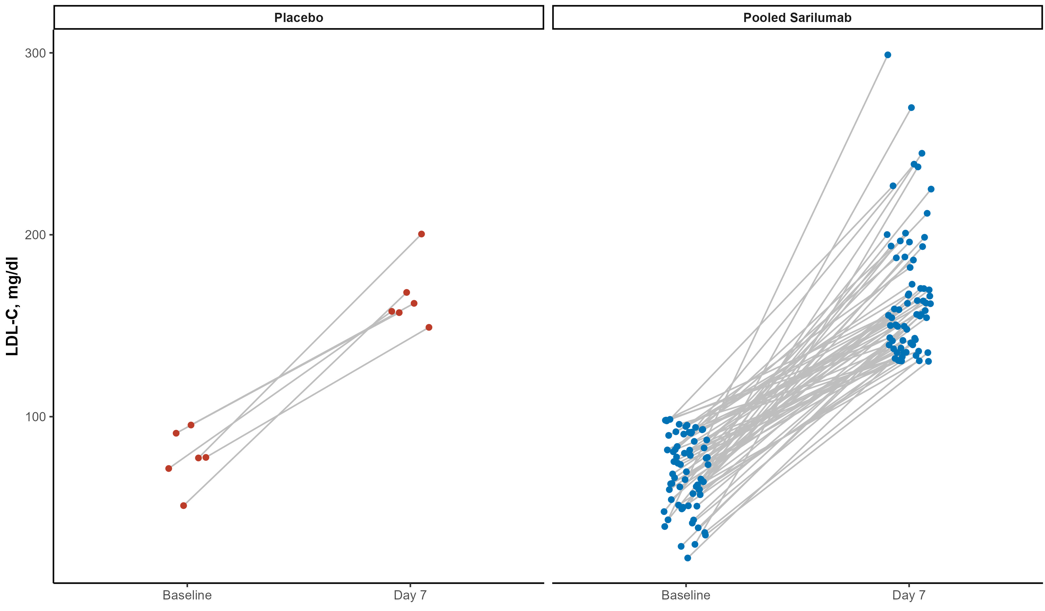


^a^Analyses of LDL-C by clinically meaningful threshold included patients with LDL-C <100 mg/dl at baseline that increased to >130 mg/dl at day 7, and those whose LDL-C was <70 mg/dl at baseline that increased to >130 mg/dl at day 7.

IQR, interquartile range.

Supplemental Figure 3. Change in lipids from baseline to day 7 by viral load at baseline


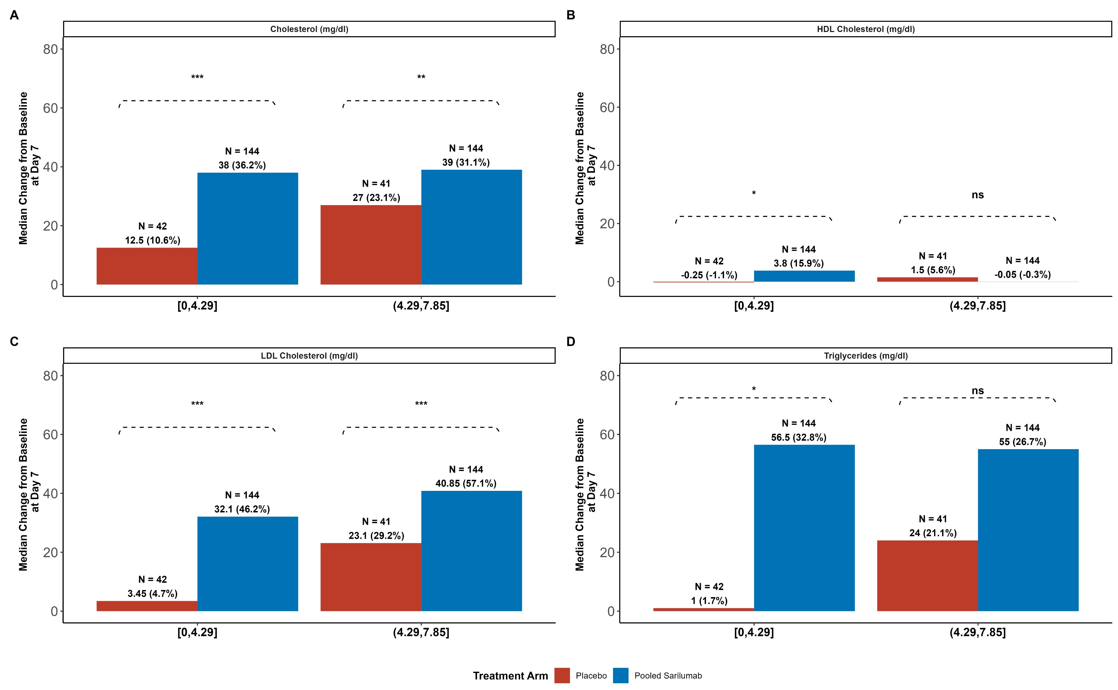


| **Lipid parameter** | **Viral load^a^** | **Median lipid value at baseline, mg/dl** | |
| --- | --- | --- | --- |
|  |  | **Placebo** | **Sarilumab** |
| TC | Low | 119.0 | 115.00 |
|  | High | 113.0 | 117.00 |
| HDL-C | Low | 24.3 | 24.70 |
|  | High | 23.8 | 25.20 |
| LDL-C | Low | 73.4 | 64.75 |
|  | High | 62.1 | 66.05 |
| TG | Low | 193.5 | 178.00 |
|  | High | 171.0 | 182.50 |

^a^Low viral load defined as 0*–*4.29 log (copies/ml); high viral load defined as 4.29*–*7.85 log (copies/ml).

TC, total cholesterol; TG, triglyceride.
